# Esophageal Pseudomelanosis Causing Pseudoachalasia

**DOI:** 10.14309/crj.0000000000001022

**Published:** 2023-04-10

**Authors:** Aaron G. Issac, Wei Zheng, Anand Jain

**Affiliations:** 1 Department of Medicine, Emory University School of Medicine, Atlanta, GA; 2 Department of Pathology, Emory University School of Medicine, Atlanta, GA; 3 Division of Digestive Diseases, Emory University School of Medicine, Atlanta, GA

## CASE REPORT

**Figure 1.**
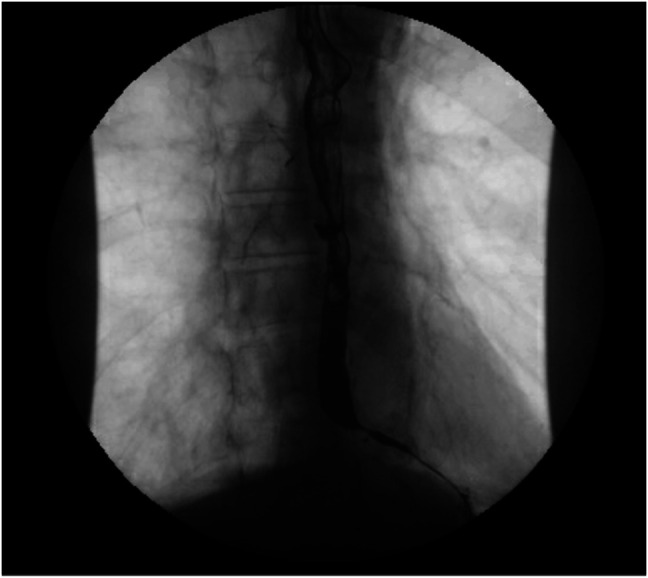
Barium esophagram demonstrating narrowing at the gastroesophageal junction.

**Figure 2.**
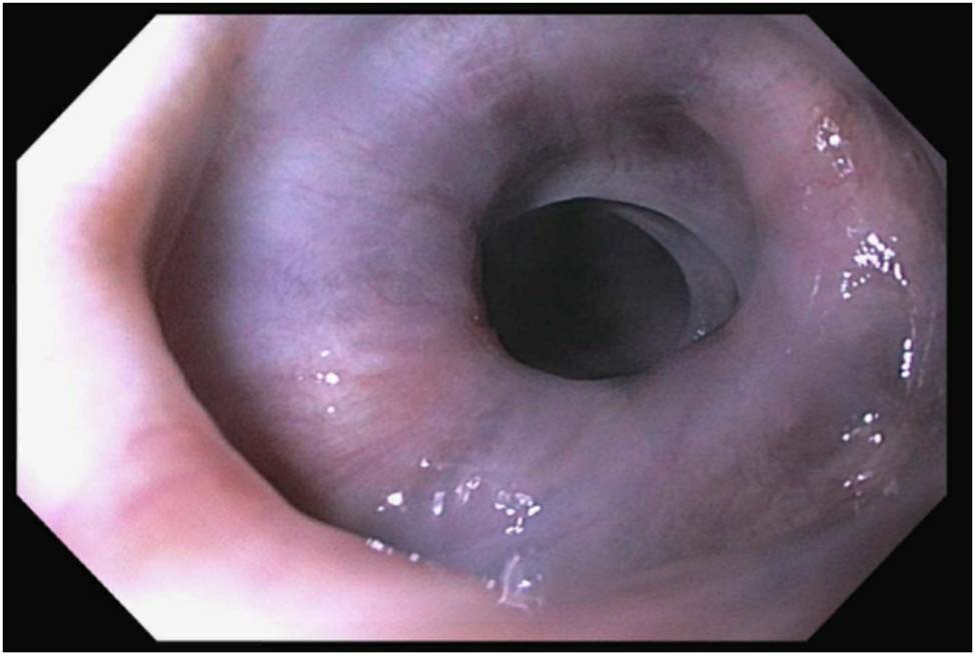
Hyperpigmentation of the distal esophageal.

**Figure 3.**
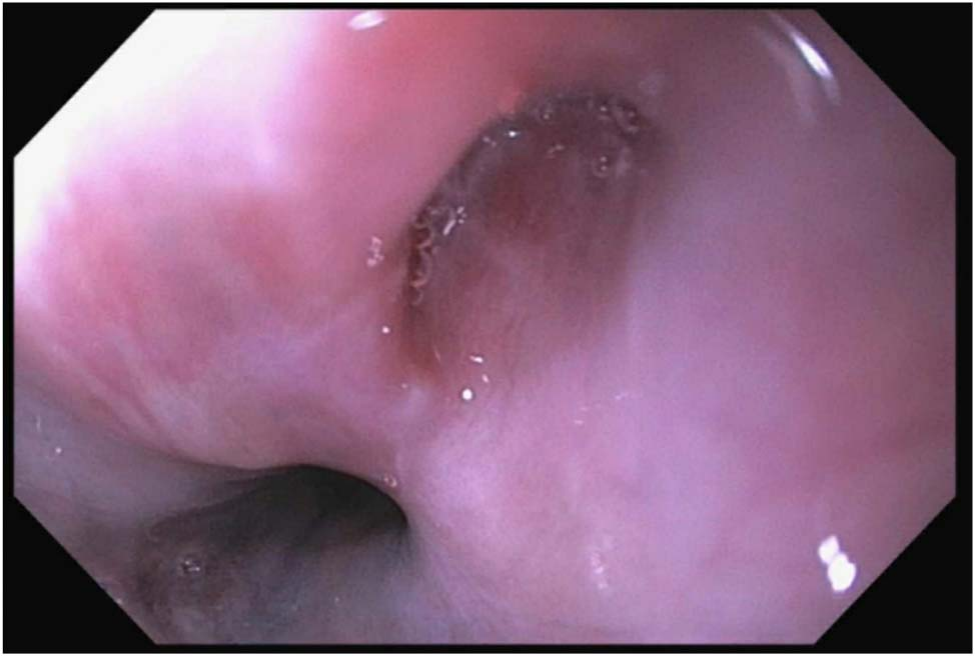
Two small diverticula in the distal esophagus.

**Figure 4.**
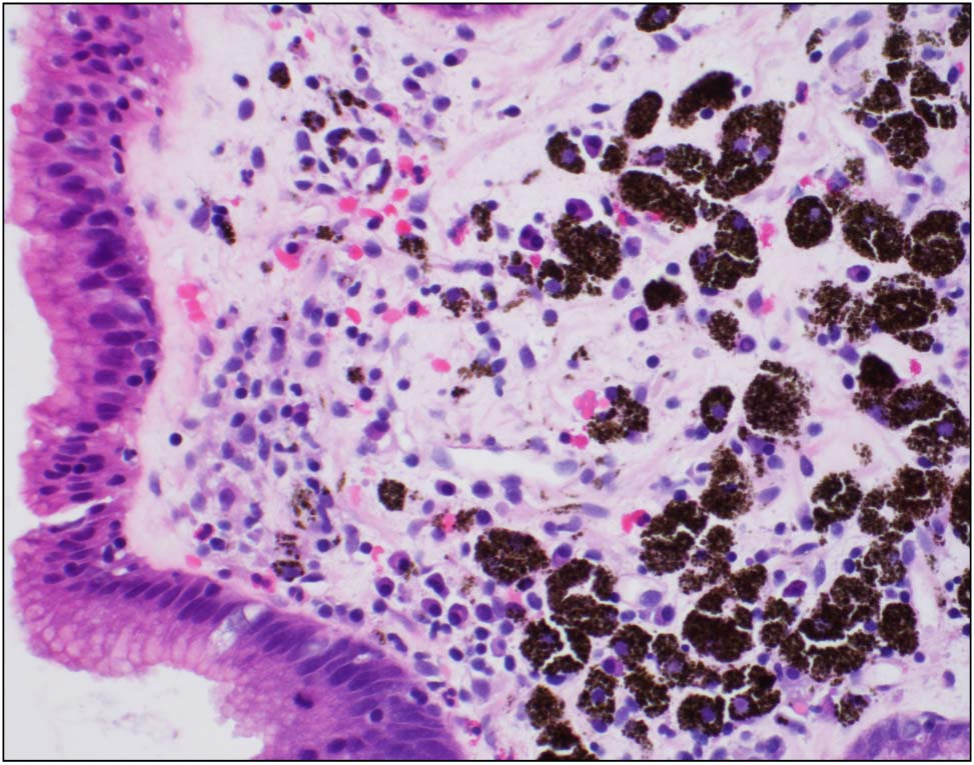
Extensive pseudomelanosis with dark brown granular pigments within histiocytes without melanocytic infiltration with hematoxylin and eosin stain.

An 86-year-old White man with a medical history of hypertension managed with hydralazine and dementia presented to our center for further evaluation of dysphagia complicated by weight loss. The patient was diagnosed 6 years ago with achalasia through a barium esophagram and received Botox injections every 6 months. His last esophagogastroduodenoscopy, 6 months before presenting to our center, reported achalasia, and he received balloon dilation and a Botox injection at the lower esophageal sphincter (LES). Esophageal manometry placement was not tolerated. Subsequent evaluation through a barium esophagram showed dysmotility (Figure 1), and endoscopy showed hyperpigmentation (Figure 2), diverticula (Figure 3), and a severely tight LES with a focal stricture but no mass. Biopsies of the distal esophagus demonstrated that the hyperpigmentation was due to dark granules without melanocytes (Figure 4). The patient was diagnosed with pseudoachalasia given endoscopic and esophagram findings of a tight LES with pseudomelanosis on histology. There are reported cases of hydralazine causing pseudomelanosis duodeni, although the patient had minimal response to stopping hydralazine.^1^ He was managed with serial dilations with an initial endoscopy balloon dilation from 8 to 12 mm, repeat dilation 1 month later from 8 mm to 15, and 4 months later dilation to 16.5 mm.

Esophageal melanocytosis describes hyperpigmentation due to melanocytic proliferation, although pseudomelanosis involves pigmented macrophages without melanocytes.^2^ Pseudomelanosis has been described in the stomach and small intestine due to chronic medical conditions, such as renal disease, diabetes, and antihypertensive drugs, such as hydralazine and furosemide.^3^ Treatment is focused on the underlying disorder, cessation of potential causative medications, and symptom management. Pseudomelanosis in the duodenum is the most common location, and the hyperpigmentation is often attributed to iron deposition. This leads to proposed mechanisms involving iron and sulfur deposition in the mucosa secondary to either medication use or impeded clearance.^4^ Esophageal pseudomelanosis is rare with only case reports in the literature and no clear etiology; it has never been described to cause strictures or pseudoachalasia. Further studies are needed to evaluate esophageal pseudomelanosis and its clinical significance.

## DISCLOSURES

Author contributions: All authors reviewed and approved the final manuscript. A. Jain: study concept and design, revision of the manuscript, and is the article guarantor. AG Issac: acquisition of data and drafting of the manuscript. W. Zheng: pathology image acquisition.

Financial disclosure: None to report.

Informed consent was obtained for this case report.
